# Chylous Ascites Following Laparoscopic Donor Nephrectomy: A Case Report

**DOI:** 10.7759/cureus.38416

**Published:** 2023-05-02

**Authors:** Omar Buksh, Abdullah M Almalki, Anfal Jar, Hani Alzahrani, Hussam Bitar, Mahmoud Al-Akraa

**Affiliations:** 1 Department of Urology, King Faisal Specialist Hospital and Research Centre, Jeddah, SAU; 2 Department of General Surgery, King Faisal Specialist Hospital and Research Centre, Jeddah, SAU

**Keywords:** ascites, renal transplant, chylous ascites, donor nephrectomy, urology

## Abstract

Chylous ascites is a form of peritoneal fluid accumulation that can arise from trauma or lymphatic obstruction. In this report, we present the first case of chylous ascites following laparoscopic donor nephrectomy in our high-volume kidney transplant center. The patient presented to the emergency department three weeks post-procedure with complaints of abdominal distention and discomfort, accompanied by constipation and nausea. Radiological confirmation of ascites was followed by paracentesis, which yielded 20 mL of milky fluid that was analyzed and confirmed as chylous ascites. A subsequent pigtail drain was inserted, resulting in a total drainage of 4 L of fluid. Chylous ascites is a rare complication of abdominal surgeries, with higher body mass index and the American Society of Anesthesiologists physical status score system being significant risk factors. Conservative management involving diet modification is the initial therapy, with percutaneous drainage or more aggressive surgical interventions considered if conservative measures are not effective, with high success rates reported for these interventions. Here, we report a case of chylous ascites following donor nephrectomy as the first case reported from our region.

## Introduction

Chylous ascites is characterized by the leakage of milky chyle, which is rich in triglycerides, into the peritoneal cavity. This condition can occur de novo as a result of trauma or obstruction of the lymphatic system, or it can develop as a secondary event in an existing clear ascitic fluid [1,2]. Chylous ascites is a rare condition with an incidence of 0.013% post-donor nephrectomy. Multiple etiological factors exist, ranging from malignancy and cirrhosis to less common causes such as postoperative complications [1]. The initial disruption of lymphatic channels transporting chyle occurs due to occlusion or trauma, leading to the accumulation of chyle in the peritoneal cavity. If left untreated, this condition can have serious consequences, especially in immune-deficient patients due to continuous loss of fat and protein-rich chyle [2].

Conservative therapy is usually the first line of treatment and focuses on improving nutritional status with a high-protein, low-fat diet and medium-chain triglycerides. Total parenteral nutrition (TPN) may also be used. Paracentesis may be considered both a diagnostic and therapeutic solution for patient symptoms and discomfort; however, repeating it is associated with multiple drawbacks such as electrolyte disturbances and serious infections. In cases of recurrence or if conservative treatment is refractory, surgical approaches such as ligation of the lymphatic can be beneficial [3].

Over the past 10 years, there has been a significant rise in the utilization of laparoscopic living donor nephrectomy procedures. This technique has become one of the most frequently performed surgeries due to its favorable outcomes, including reduced postoperative complications, decreased pain, shorter hospital stays, and improved cosmetic appearance when compared to open surgery. Despite some reported postoperative complications, chylous ascites is among the least frequently reported [4]. While this condition has been rarely reported in several countries, this is the first documented case from our region.

## Case presentation

A 43-year-old female presented for a pre-transplant workup for donor nephrectomy to her husband, with a known history of hypothyroidism and currently taking levothyroxine at a dose of 50 µg per day. Additionally, she reported well-controlled bronchial asthma, with a diagnosis of latent tuberculosis during the transplant workup. She was started on an isoniazid course and subsequently cleared by the transplant infectious disease team. Notably, her body mass index (BMI) was 34.7 kg/m^2^. The patient had a left kidney with two renal arteries and two renal veins that communicated in one vein before joining the inferior vena cava. Following clearance, she underwent a laparoscopic donor left nephrectomy. The procedure went smoothly with minimal blood loss, and her postoperative recovery was uneventful. Her Foley catheter was removed the day after surgery, and she remained stable with normal vital signs, abdominal examination, bowel movements, and blood work. She was discharged home in good condition on postoperative day two.

**Figure 1:**
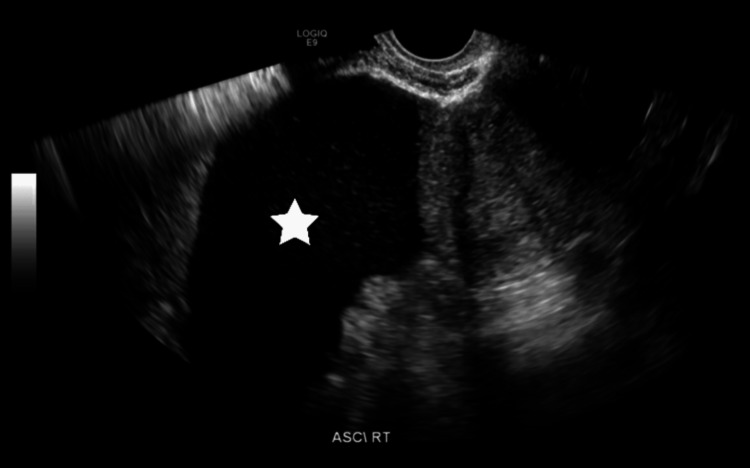
An ultrasound of the abdomen showing moderate free intra-abdominal fluid on the right lower side.

**Figure 2:**
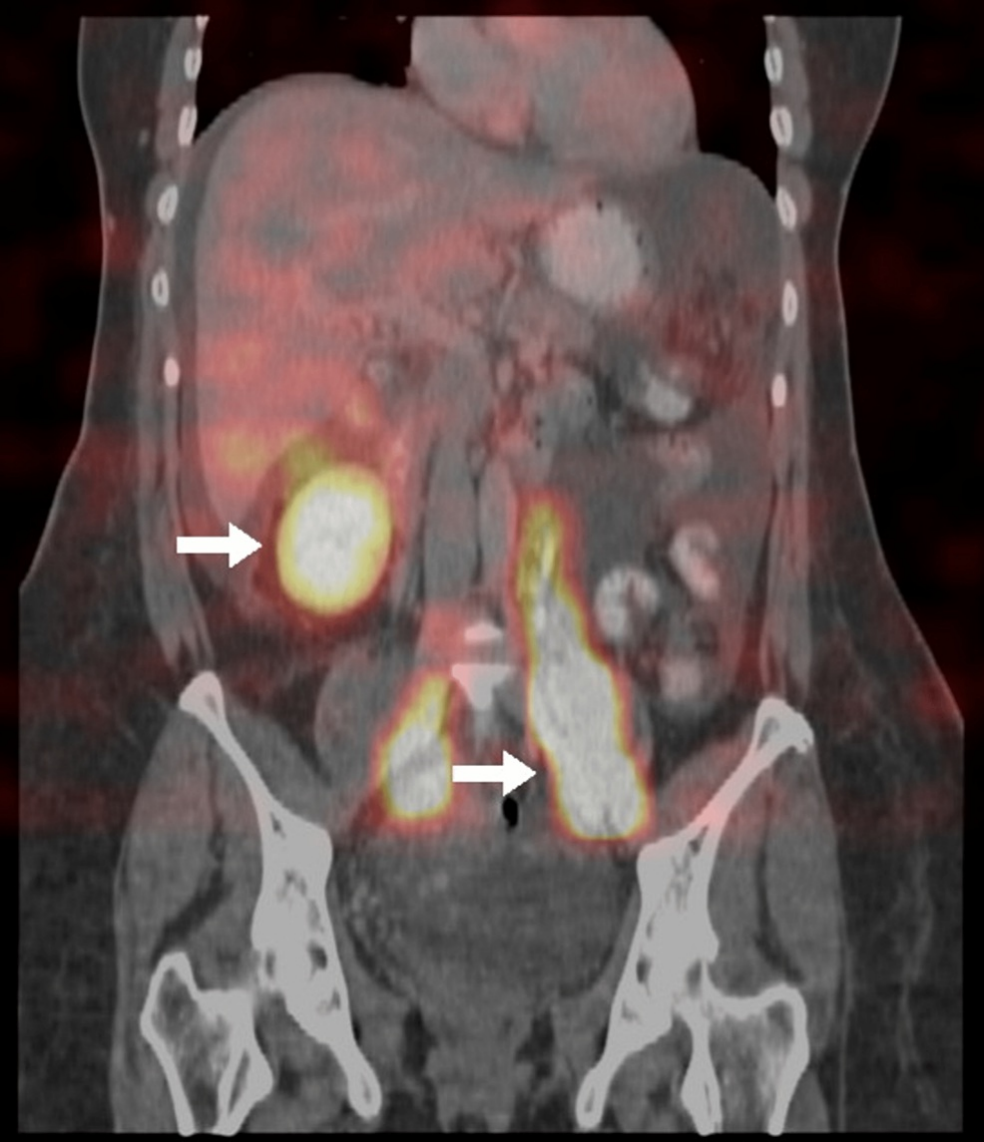
Nuclear lymphangiogram showing lower pelvic tracer accumulation consistent with chyle ascites, in addition to right kidney chyluria.

Three weeks later, the patient presented to the emergency room complaining of abdominal distention that gradually developed over the course of one week. She also reported constipation and nausea but denied fever. At the time of admission under urology care (her surgeon being a urology and kidney transplant specialist), she remained vitally stable and afebrile with normal blood work and electrolyte levels. An ultrasound of her abdomen revealed a moderate amount of intra-abdominal fluid in the right lower abdomen (Figure 1). Abdominal paracentesis was performed, resulting in the initial aspiration of 20 mL of milky fluid. Subsequently, an abdominal drain was inserted by an interventional radiologist. Based on the fluid analysis from the drain, which revealed a cholesterol level of 4.4 mmol/L, triglyceride level of 19.4 mmol/L, protein level of 55 g/L, creatinine level of 67 µmol/L, and negative for chylomicrons, the patient was diagnosed with chylous ascites. Additionally, the patient’s serum triglyceride was found to be 9.9 mmol/L and serum cholesterol was 2.8 mmol/L. A nuclear lymphangiogram was performed, which showed a lower pelvic tracer accumulation consistent with chyle ascites and right kidney chyluria (Figure 2).

Initially, conservative management was planned for the patient and included nil per os status and TPN with diet modification for five days. The patient was also started on metronidazole and broad-spectrum antibiotics. Over the next few days, the patient’s condition improved significantly with a resolution of abdominal pain and nausea. The pigtail drainage yielded a total output of approximately 4 L and abdominal distention improved remarkably until it resolved over the next five days.

Upon discharge from the hospital, the patient was in good condition with the abdominal drain still in place. In addition, the patient was instructed to adhere to a fat-free, high-protein diet with salt restriction. After one week during a follow-up clinic visit where there had been no output from the drain for several days prior, it was removed.

## Discussion

Chylous ascites is a medical condition that arises from either a lymphatic blockage or intraoperative trauma-induced leakage [3]. This benign disease is typically managed conservatively in the majority of cases, with a success rate of approximately 93.6% [5]. Chylous ascites has been reported in various surgeries, particularly those involving extensive retroperitoneal dissection, such as abdominal aortic surgery (0%-1%) and retroperitoneal lymph node dissection (2%-7%) [6]. Although rare, laparoscopic nephrectomy has also been associated with chylous ascites, with an incidence rate of 0.013% [1]. While higher BMI has been identified as a contributing factor in some studies, the American Society of Anesthesiologists (ASA) physical status score has been found to be the most significant risk factor for this complication. However, it was reported by Kim et al. that BMI and previous abdominal surgeries were not significantly associated with an increased risk of chylous ascites [7]. The ASA score of our patient was 2, with a BMI of 34.7 kg/m^2^.

Conservative management through nutritional modification has shown remarkable success rates, including salt restriction, low-fat and high-protein diets, and medium-chain triglyceride diets. TPN may be initiated if conservative measures fail; however, somatostatin and TPN therapy may also be considered as first-line treatment in some cases [2]. In rare instances where conservative measures are ineffective, surgical intervention through direct ligation of the leaking lymphatics may be attempted with high success rates reported [8].

The management of chylous ascites involves evolving timing and rules for surgical intervention. A case series conducted by Aerts et al. found that seven out of 18 patients eventually required surgery for treatment [6]. Surgical intervention is necessary when lymphoembolization is unsuccessful and involves identifying the leaking lymphatic and either clipping or suturing it or using fibrin glue to seal it [6,9-11]. The appropriate timing for surgical intervention is a topic of controversy, with previous studies recommending conservative treatment for 4-12 weeks before considering surgery [12-15].

We considered several possible diagnoses, such as retroperitoneal hematoma, stump bleeding, and seroma, but ultimately suspected chylous ascites due to the patient’s procedure, which was confirmed by fluid analysis and lymphangiogram. The left-sided donor nephrectomy procedure involves ligating the right renal artery near the aorta, which can damage the para-aortic lymphatic vessels and lead to chylous ascites [16].

## Conclusions

Chylous ascites is an extremely rare complication of abdominal surgeries, especially post-donor nephrectomy, with higher BMI and ASA physical status score being important risk factors. Diagnosis is typically based on the analysis of the accumulated fluid. Treatment is usually conservative, as in our case, with surgical intervention still valid in rare instances.
